# A new strategy to fight antimicrobial resistance: the revival of old antibiotics

**DOI:** 10.3389/fmicb.2014.00551

**Published:** 2014-10-20

**Authors:** Nadim Cassir, Jean-Marc Rolain, Philippe Brouqui

**Affiliations:** ^1^Unité de Recherche sur les Maladies Infectieuses et Tropicales Emergentes, UM63 CNRS 7278 IRD 198 INSERM U1095, Facultés de Médecine et de Pharmacie, Aix-Marseille UniversitéMarseille, France; ^2^Institut Hospitalo-Universitaire en Maladies Infectieuses et Tropicales, Hôpital Nord, Assistance Publique - Hôpitaux de MarseilleMarseille, France

**Keywords:** multi-drug-resistant pathogens, MRSA and VRE, ESBLs, colistin, fosfomycin, trimethoprim-sulfamethoxazole combination, tetracycline, MDR-tuberculosis

## Abstract

The increasing prevalence of hospital and community-acquired infections caused by multidrug-resistant (MDR) bacterial pathogens is limiting the options for effective antibiotic therapy. Moreover, this alarming spread of antimicrobial resistance has not been paralleled by the development of novel antimicrobials. Resistance to the scarce new antibiotics is also emerging. In this context, the rational use of older antibiotics could represent an alternative to the treatment of MDR bacterial pathogens. It would help to optimize the armamentarium of antibiotics in the way to preserve new antibiotics and avoid the prescription of molecules known to favor the spread of resistance (i.e., quinolones). Furthermore, in a global economical perspective, this could represent a useful public health orientation knowing that several of these cheapest “forgotten” antibiotics are not available in many countries. We will review here the successful treatment of MDR bacterial infections with the use of old antibiotics and discuss their place in current practice.

## Introduction

Antimicrobial resistance is one of the greatest threats to human health worldwide (Walker et al., 2009). It dramatically reduces the probability of effectively treating infections and increases the morbidity and mortality associated with common bacterial diseases (Klevens et al., 2007). Since the discovery of penicillin in 1928, antimicrobial resistance has been linked to antibiotic use. Recent studies of bacteria in permafrost samples documented the existence of resistance genes 30,000 years ago (D'Costa et al., 2011), emphasizing that antibiotic use and misuse favor resistance through selection pressure (Rolain et al., 2013). It has been demonstrated that antimicrobial resistance prevalence can be diminished through decreased antibiotic consumption (Seppälä et al., 1997). This complex ecological phenomenon depends on individuals, bacterial strains, and mechanisms of resistance (Andersson and Hughes, 2010). Old and new antibiotics vary in their impact on the emergence and spread of resistant bacteria (Sullivan et al., 2001).

Bacterial strains resistant to newly developed antibiotics have emerged recurrently (Long and Vester, 2012). Therefore, antimicrobial resistance presents an ongoing challenge that requires a multifaceted approach including (i) biomedical innovation, (ii) improved surveillance of antibiotic consumption and antimicrobial-resistance rates, (iii) prevention of health-care-associated infections and transmission of multidrug-resistant (MDR) bacteria and environmental dissemination, (iv) rapid microbiological diagnosis, and (v) curtailed clinical and veterinary misuse. It is alarming that although bacterial resistance continues to emerge, the rate at which antibiotics are being developed is decreasing. In this context, the reintroduction of previously used antibiotics active against MDR bacteria represents a new alternative for the control of antimicrobial resistance (Pulcini et al., 2012). As old antibiotics have rarely been subjected to contemporary drug-development procedures or compared to commonly used antibiotics, they are less considered in practice guidelines. Therefore, their efficacy and safety must be revaluated to optimize therapy.

This review aims at providing a non-exhaustive collection of microbiological and clinical data on potentially useful older antibiotics. We consider the value of “forgotten” antibiotics for the treatment of (i) MDR Gram-negative bacterial infections (polymyxins, fosfomycin, mecillinam, temocillin, and nitrofurantoin); (ii) MDR Gram-positive infections [trimethoprim-sulfamethoxazole (TMP/SMX), tetracyclines, chloramphenicol, clindamycin, pristinamycin, rifampicin, and fusidic acid]; and (iii) MDR tuberculosis (clofazimine, amoxicilline-clavulanate, TMP/SMX, and minocycline).

## Forgotten antibiotics for multidrug-resistant gram-negative bacteria

The incidence of infections caused by MDR Gram-negative bacteria has increased worldwide over the last decade. The majority of *Escherichia coli* and *Klebsiella pneumoniae* isolates reported by European Antimicrobial Resistance Surveillance Network (EARS-Net) in 2012 were resistant to at least one of the antimicrobials tested, and many had combined resistance to third-generation cephalosporins, fluoroquinolones, and aminoglycosides (http://www.ecdc.europa.eu). Of particular importance to public health is the spread beta-lactamases with an extended spectrum, which confer resistance to most β-lactams (even carbapenems for some of them) and are frequently associated with resistance to other groups of antibiotics in isolates of Enterobacteriaceae both from community and health-care settings (Pitout and Laupland, 2008; Zahar et al., 2009; Diene et al., 2013). In this context, it is essential to optimize our antibacterial armamentarium. One major step is to reconsider currently active and available antibiotics. Table 1 summarizes the successful clinical treatment of infections caused by MDR Gram-negative bacteria with “forgotten” antibiotics, either alone or in combination.

**Table 1:** **Infections caused by multidrug-resistant Gram-negative bacteria successfully treated with old antibiotics**.

**Molecule**	**Pathogens**	**Sites of infection**	**References**
Colistin (IV)	MDR *A. baumannii* MDR *P. aeruginosa* MDR *K. pneumonia* MDR *S. maltophilia*	VAP HA-pneumonia UTI IAI BJI Bacteremia Wound infection Meningitis PJI Diabetic foot infection	Jiménez-Mejías et al., 2002; Garnacho-Montero et al., 2003; Linden et al., 2003; Markou et al., 2003; Fulnecky et al., 2005; Kasiakou et al., 2005a,b; Falagas et al., 2006a,b; Motaouakkil et al., 2006; Tascini et al., 2006; Kallel et al., 2007; Koomanachai et al., 2007; Rios et al., 2007; Bassetti et al., 2008; Betrosian et al., 2008; Mastoraki et al., 2008; Song et al., 2008; Taşbakan et al., 2009; Kofteridis et al., 2010
Fosfomycin (IV)	ESBL *E. coli* ESBL *K. pneumonia*/Enterobacter sp./Serratia sp. MDR *P. aeruginosa* OXA-48 *K. pneumonia* and *E. coli* KPC *K. pneumonia* Carbapenem-resistant *P. aeruginosa* MDR *S. enterica* serotype Typhimurium	VAP HA-pneumonia UTI IAI BJI Bacteremia Wound infection Meningitis Brain abscess Lung abscess Cystic fibrosis (pulmonary exacerbation)	Mirakhur et al., 2003; Nakaya et al., 2003; Michalopoulos et al., 2010; Apisarnthanarak and Mundy, 2012; Dinh et al., 2012; Karageorgopoulos et al., 2012; Navarro-San Francisco et al., 2013; Pontikis et al., 2014
Fosfomycin (PO)	ESBL *E. coli* and *K. pneumoniae* KPC *K. pneumoniae* MDR *P. aeruginosa*	Lower UTI	Pullukcu et al., 2007; Senol et al., 2010; Neuner et al., 2012
Pivmecillinam Mecillinam (PO)	ESBL *E. coli* and *K. pneumonia* CTX-M/ESBL *E. coli* ESBL Enterobacteriaceae	Lower UTI Relapsing pyelonephritis Complicated UTI	Nicolle and Mulvey, 2007; Titelman et al., 2012; Jansåker et al., 2014; Søraas et al., 2014
Temocillin (IV)	dAmpC/ESBL Enterobacteriaceae ESBL *E. coli* and *K. pneumonia* MDR *P. agglomerans*	HA pneumonia UTI Bacteremia Severe sepsis (VAP, UTI, IAI) Epidural abscess Subacute synovitis	Barton et al., 2008; Duerinckx, 2008; Gupta et al., 2009; Balakrishnan et al., 2011
Nitrofurantoin (PO)	ESBL *E. coli*	Lower UTI	Tasbakan et al., 2012

*MDR, Multidrug-resistant; P. aeruginosa, Pseudomonas aeruginosa; A. baumanii, Acinetobacter baumanii; E. coli, Escherichia coli; K. pneumonia, Klebsiella pneumoniae; S. enterica, Salmonella enterica; P. agglomerans, Pantoea agglomerans; VAP, Ventilator-associated pneumonia; HA, Hospital-acquired; UTI, Urinary-tract infection; BJI, Bone and joint infection; PJI, Prosthetic-joint infection; IAI, Intra-abdominal infection; ESBL, Extended-spectrum β-lactamases; IV, Intravenous; PO, Per os*.

### Polymyxins

Colistin, synthesized by *Paenibacillus polymyxa* subspecies *colistinus*, was discovered in 1949 (Komura and Kurahashi, 1979). Polymyxin B and polymyxin E (colistin) are the 2 polymyxins used in clinical practice. Their mechanism of action includes attachment to the outer cell membrane of Gram-negative bacteria, leading to membrane-permeability changes and cell death. Colistin was initially used in Japan and in Europe during the 1950s and was used in the United States in the form of colistimethate sodium in 1959 (Reed et al., 2001). Intravenous formulations of colistin and polymyxin B were gradually abandoned in most parts of the world in the early 1980s because of the reported high incidence of nephrotoxicity (Biswas et al., 2012). Indeed, most of the drug is excreted in the urine, and dose adjustment is required in patients with renal failure. Recent studies have shown that better management of intravenous formulations led to considerably less toxicity than was reported in old studies (Falagas and Kasiakou, 2005). Inhaled colistin has been proposed as alternative, in adjunction to systemic therapy or alone, for the treatment of pneumonia or chronic lung infection with MDR Gram-negative bacteria (Lu et al., 2012; Tumbarello et al., 2013). Although promising results have been reported, more data are needed to establish its appropriate role in mechanically-ventilated patients (Michalopoulos and Falagas, 2014).

Colistin was recommended by the most recent American Thoracic Society Guidelines as a therapeutic option for the treatment of ventilator-associated pneumonia (VAP) caused by MDR Gram-negative organisms (American Thoracic Society, and Infectious Diseases Society of America, 2005). Intravenous polymyxins has been evaluated for the treatment of serious MDR *P. aeruginosa, Acinetobacter baumannii* and Enterobacteriaceae infections of various types, including pneumonia, bacteremia, abdominal infections, bone and joint infections (BJIs), urinary-tract infections (UTIs), and meningitis (Falagas and Kasiakou, 2005) (Table 1).

One worrisome problem is the emergence of colistin resistance in KPC-producing bacteria, as seen in up to 20% of isolates in some countries (Kontopidou et al., 2014). The spread of colistin resistance may be related to the extensive use of this drug in empiric and targeted treatment and also due to the ecological effect of topical use in selective decontamination protocols in intensive-care units (Oostdijk et al., 2013). But, the most common mechanism of colistin resistance is modification of LPS (Park et al., 2011). As it chromosomal, it is not prone to spread horizontally, but relies exclusively on clonal expansion. Several other mechanisms have been suggested, most of which involve changes in the outer membrane (Li et al., 2006). *Paenibacillus polymyxa* subspecies *colistinus*, also produces colistinase, which inactivates colistin. However, enzymatic resistance of bacteria to colistin has not been reported in clinical practice (Falagas and Kasiakou, 2005). Interestingly, decades of colistin use in veterinary medicine has not been associated with an increased resistance prevalence in commensal *Escherichia coli* isolated from animals (Wasyl et al., 2013). Regarding *Acinetobacter baumanii*, colistin resistance has emerged worldwide (Cai et al., 2012). Although it associated with impaired virulence (Rolain et al., 2011), combination therapy might be the best antimicrobial strategy. Besides, optimization of colistin use should prevent the emergence of species naturally resistant to colistin, such as *S. marcescens* and *P. mirabilis*, which have the potential to cause outbreaks (Merkier et al., 2013).

### Fosfomycin

Fosfomycin is an antimetabolite inhibitor that prevents the formation of N-acetylmuramic acid, an essential precursor of peptidoglycan-chain formation in the bacterial wall. It was first identified in Spain in 1969 in the fermentation broths of several strains of *Streptomyces* sp. (Raz, 2012). Fosfomycin has a broad spectrum of antimicrobial activity, including a rapid bactericidal effect against several Gram-negative and Gram-positive aerobic bacteria (Falagas et al., 2009). Unlike many alternatives, fosfomycin formulations are generally well tolerated, with minimal toxicity (excepting thrombophlebitis when administered via peripheral venous catheter).

The use of intravenous fosfomycin formulations for the treatment of infections caused by MDR bacteria has shown a high rate of clinical success (Table 1). For lower UTIs, the successful use of oral fosfomycin tromethamine is well documented.

One of the first studies on the broad use of fosfomycin in Spain noted development of resistance in 3% of the 959 cases in total but in 10% of the 86 *P. aeruginosa* infections (Rodríguez et al., 1977). These rates of emergence appear to be stable over time in countries where fosfomycin use has been used for decades (Falagas et al., 2008). But, fosfomycin resistance is in many bacterial species caused by plasmid-mediated fosfomycin genes (Suárez and Mendoza, 1991). Currently, plasmid-mediated fosfomycin resistance determinants, have been discovered and are related to fosfomycin resistance in *Escherichia coli* (*fosA, fosA3, fosC*) (Lee et al., 2012), *Enterobacter cloacae* (*fosA2*) (Xu et al., 2011), *Klebsiella pneumoniae* (*fosA, fosA3*) (Lee et al., 2012), *Staphylococcus* spp. (*fosB*) (Suárez and Mendoza, 1991), and, *Enterococcus faecium* (*fosB3*) (Xu et al., 2013). Chromosomally resistant mutants have also been well described (Suárez and Mendoza, 1991). This is a major concern about the potential for the selection and spread of resistance. Then, when used to treat systemic infections, intravenous fosfomycin may be a component of combination therapy administered (Pogue et al., 2011).

### Pivmecillinam/mecillinam

Pivmecillinam is the prodrug of mecillinam, a unique β-lactam with high specificity against penicillin-binding protein 2 (PBP-2) in the Gram-negative cell wall and therefore is highly active against Enterobacteriaceae and resistant to β-lactamases. This drug for oral use is well tolerated and can be given in patients with impaired renal function, but its usefulness is limited by its lack of activity against Gram-positive organisms and *Pseudomonas aeruginosa* (Dewar et al., 2014). Since its introduction in the 1970s, it has been widely used for the treatment of acute lower UTIs, primarily in the Nordic countries (Graninger, 2003).

Treatment of lower UTIs caused by ESBL-producing *E. coli* has produced variable results (Titelman et al., 2012; Poulsen et al., 2013; Søraas et al., 2014) (Table 1). The high rates of treatment failure (44%) reported by Søraas et al. raised concerns regarding the optimal dosing that suggest the need for further studies (Søraas et al., 2014). Treatment with pivmecillinam was successful in a case of relapsing pyelonephritis caused by ESBL-producing *E. coli* where other treatments had failed (Nicolle and Mulvey, 2007). Currently, the use of pivmecillinam as first-line treatment for uncomplicated UTIs is recommended by the Infectious Diseases Society of America and the European Society for Microbiology and Infectious Diseases (Gupta et al., 2011).

The widespread and long-term use of pivmecillinam for UTIs in Scandinavian countries is well documented, with no indication of any significant increase in mecillinam resistance in these countries (Graninger, 2003). Indeed, Pivmecillinam is a prodrug and thus has a low impact on the endogenous microflora (Sullivan et al., 2001). Although poorly studied, mecillinam resistance have been shown to be caused by mutations in the genes affecting the elongation process of the bacteria, and associated with substantial fitness cost *in vitro* to the resistant *E. coli* (Wachi et al., 1987). These properties lead to a decreased potential for the selection of resistance.

### Temocillin

Developed and first marketed in the UK in the 1980s, temocillin was quickly abandoned because its lack of activity against Gram-positive organisms, anaerobes and *Pseudomonas aeruginosa* was perceived as a major limitation (Slocombe et al., 1981). This beta lactam antibiotic, that is the 6-α-methoxy derivative of ticarcillin, is also characterized by its resistance to most beta-lactamases with an extended spectrum (i.e., TEM, SHV, CTX-M, AmpC) and some carbapenemases (Livermore et al., 2006; Rodriguez-Villalobos et al., 2006; Glupczynski et al., 2007; Adams-Haduch et al., 2009). Temocillin is mainly excreted renally and therefore requires dosage adjustment in patients with renal impairment.

Based on temocillin's relatively narrow spectrum, it is appropriate for use in microbiologically directed therapy, particularly for the UTIs caused by confirmed ESBL producers. In this setting, temocillin might be used as an alternative to carbapenems (Livermore and Tulkens, 2009), as temocillin is ecologically safer. Research in Belgium suggests that temocillin is also effective in severe infections such as VAP if the organism appears susceptible *in vitro* (De Jongh et al., 2008). It is also a potential alternative treatment option for mild-to-moderate UTIs caused by KPC-producing Enterobacteriaceae (Adams-Haduch et al., 2009).

The first reported case of breakthrough bacteremia during temocillin treatment occurred in a patient with renal dysfunction and probable insufficient dosing (1 g once daily) (Gupta et al., 2009). NDM-1 and OXA-48 carbapenemases also confer resistance to temocillin (Livermore et al., 2011). Although poorly studied, the main mechanisms of resistance to temocillin are efficient efflux or reduced PBP binding that could explain high MICs to *Pseudomonas aeruginosa* (Livermore and Tulkens, 2009).

### Nitrofurantoin

Nitrofurantoin (NFT), which is a synthetic antimicrobial derived from furan by the addition of a nitro group and a side chain containing hydantoin, was introduced into clinical practice in its microcrystalline form in 1952. Since the macrocristalline form was developed, the previously frequent gastrointestinal side effects have become less common, giving this drug a good safety profile (Cunha, 1989). This molecule has a broad-spectrum activity against the main uropathogens (i.e., *Escherichia coli, Citrobacter* species, group-B streptococci, enterococci, *Staphylococcus aureus, S. epidermidis, Klebsiella pneumonia*, and *Enterobacter* sp.) and has also been shown to be active against ESBL-producing Enterobacteriaceae and vancomycin-resistant enterococci. Notably, it lacks activity against *Pseudomonas aeruginosa, Serratia marscecens*, and *Proteus mirabilis* (Cunha et al., 2011).

NFT is effective both *in vitro* and in clinical studies against ESBL-producing *E. coli* (Table 1); however, given that patients with acute pyelonephritis respond inconsistently to NFT and that bacteremia has occurred during NFT treatment, its use has been restricted to lower UTIs (Tasbakan et al., 2012). Currently, NFT is recommended as a first-line treatment for uncomplicated UTIs by the Infectious Diseases Society of America and the European Society for Microbiology and Infectious Diseases (Gupta et al., 2011).

Recent surveys have found a persistent low prevalence of resistance to NFT (1.9–7.7%) among urinary *E. coli* isolates, including those resistant to TMP/SMX or ciprofloxacin; the prevalence of this resistance reaches 23.2% in ESBL-producing *E. coli* (Tasbakan et al., 2012). Chromosomally resistance-conferring mutations have been identified in the *nsfA* and *nsfB* genes that encode oxygen-insensitive nitroreductases, and associated with substantial fitness cost *in vitro* to the resistant *E. coli* (Sandegren et al., 2008). These properties lead to a decreased potential for the selection of clinically relevant resistance.

## Forgotten antibiotics for multidrug-resistant gram-positive bacteria

*Staphylococcus aureus* and *Enterococcus* spp. are two of the most common organisms causing nosocomial infections and are consistently associated with high mortality rates (Pea and Viale, 2007). Resistance among these pathogens to first-line agents such as methicillin and vancomycin continues to rise, while isolates with reduced susceptibility to newer agents, including linezolid, are also emerging (Patel et al., 2013). In Table 2, we summarized the successful clinical treatment of infections caused by MDR Gram-positive bacteria with “forgotten” antibiotics alone or in combination.

**Table 2:** **Infections caused by multidrug-resistant Gram-positive bacteria successfully treated with old antibiotics**.

**Molecule**	**Pathogens**	**Sites of infection**	**References**
Trimethoprim-sulfamethoxazole (TMP/SMX) (IV, PO)	CA and HA-MRSA *E. americana*	SSTI BJI Osteomyelitis IE (prosthetic valve) Meningitis Bacteremia COPD exacerbation	Shafqat et al., 1971; Seligman et al., 1973; Bengtsson et al., 1974; Tamer and Bray, 1982; Levitz et al., 1984; Stein et al., 1998; Cenizal et al., 2007; Pound et al., 2007; Nguyen et al., 2009; Goldberg et al., 2010; Cadena et al., 2011; Messina et al., 2011; Di Carlo et al., 2013
Minocycline (IV, PO)	MRSA	SSTI BJI Osteomyelitis IE (prosthetic valve) Bacteremia	Clumeck et al., 1984; Lawlor et al., 1990; Moreno et al., 1994; Ruhe et al., 2005; Cenizal et al., 2007; Ruhe and Menon, 2007
Doxycycline (IV, PO)	MRSA VRE*f*	SSTI UTI Bacteremia	
Chloramphenicol (IV)	VRE VRE*f S. maltophilia*	Meningitis Ventriculitis Bacteremia IAI EI (prosthetic valve)	Norris et al., 1995; Lautenbach et al., 1998; Pérez Mato et al., 1999; Mehta et al., 2000; Ricaurte et al., 2001; Scapellato et al., 2005
Clindamycin (IV, PO)	MRSA MRSA (PVL+)	SSTI BJI Mandible osteomyelitis Necrotizing fasciitis Necrotizing pneumonia Bacteremia	Martínez-Aguilar et al., 2003; Hidron et al., 2009; Tobeña Rué et al., 2009; Tuzuner-Oncul et al., 2009; Kefala-Agoropoulou et al., 2010
Pristinamycin (PO)	MRSA VRE CoNS	Pneumonia SSTI UTI BJI Epidural abscess	Dancer et al., 2003; Ng and Gosbell, 2005; Ruparelia et al., 2008; Reid et al., 2010
Fusidic acid (PO)	MRSA	BJI PJI Chronic osteomyelitis Epidural abscess	Nather et al., 2005; Howden and Grayson, 2006; Aboltins et al., 2007; Murray et al., 2008; Ahamed Puthiyaveetil, 2009; Ferry et al., 2010; Chiang et al., 2011; O'Neill et al., 2011; Wolfe, 2011
Nitrofurantoin (PO)	VRE*f*	Chronic prostatitis	Taylor et al., 1998
Fosfomycin (PO)	VRE*f*	Lower UTI	Neuner et al., 2012
Fosfomycin (IV)	MRSA CoNS	Meningitis IE PJI	De Boutin et al., 1985; Roualdes et al., 1985; Silbermann et al., 1995; Lee et al., 2013

*MRSA, methicillin-resistant Staphylococcus aureus; CA, community-associated; CoNS, coagulase-negative Staphylococci; VRE, vancomycin-resistant Enterococcus sp.; VREf, vancomycin-resistant Enterococcus faecium; S. maltophilia, Stenotrophomonas maltophilia; VAP, ventilator-associated pneumonia; IE, infective endocarditis; UTI, urinary-tract infection; BJI, bone and joint infection; PJI, prosthetic-joint infection; IAI, intra-abdominal infection; IV, Intravenous; PO, Per os*.

### Trimethoprim-sulfamethoxazole

The combination drug TMP/SMX acts as a broad-spectrum bactericidal agent and was introduced clinically in the early 1970s (Grim et al., 2005). Trimethoprim is a tetrahydrofolate reductase inhibitor that, when added to sulfamethoxazole, provides a second-step block in the folate biosynthetic pathway (Proctor, 2008). According to its good oral bioavailability, high-dosage regimen of TMP/SMX represents a suitable alternative for methicillin-resistant *S. aureus* (MRSA) infections (Muhammed Ameen et al., 2014).

TMP/SMX is recommended as first-line treatment for uncomplicated UTIs, skin and soft-tissue infections (SSTIs), and community-associated methicillin-resistant *Staphylococcus aureus* (CA-MRSA) infections, according to clinical practice guidelines (Gupta et al., 2011; Stevens et al., 2014). In a recent study with a cohort of 328 patients with infections due to MRSA with a MIC to vancomycin of 2 μg/mL, TMP/SMX alone compared favorably to linezolid and daptomycin in terms of treatment efficacy, mortality, and reduced antibiotic costs (Campbell et al., 2012). Although TMP/SMX alone has been shown to be less effective than current treatment for MRSA endocarditis (i.e., vancomycin or teicoplanin) (de Górgolas et al., 1995), in combination with other antibiotics (i.e., daptomycin, clindamycin or vancomycin, and rifampicin) successful treatments have been reported (Fujino et al., 2009; Casalta et al., 2013; Di Carlo et al., 2013). Only one randomized, prospective trial compared TMP-SMZ to vancomycin for the treatment of *S. aureus* (47% MRSA) endovascular infections in injection-drug users. In this study, TMP-SMZ was inferior to vancomycin only for the treatment of methicillin-susceptible *S. aureus* (MSSA), while the cure rates and other clinical and microbiological outcomes against the MRSA group were similar for both drugs (Markowitz et al., 1992). Long-term oral ambulatory treatment with TMP-SMZ appeared to be an effective alternative to the conventional medicosurgical treatment of chronic MDR *Staphylococcus* sp.-infected orthopedic implants (Stein et al., 1998). The scarcity of data concerning TMP-SMZ treatment for *S. aureus* infections emphasizes the need for further clinical studies comparing this drug to other available options, specifically vancomycin (Adra and Lawrence, 2004).

Over a 10-year period, interrupted time-series analysis demonstrated that the CA-MRSA period was associated with a significant increase in use of TMP/SMX but showed no significant changes in the rates of susceptibility among clinical isolates. There was also no evidence for selection of organisms with increased resistance to other antimicrobials in relation to increased TMP-SMZ use (Wood et al., 2012). Moreover, a recent report has shown high rates of susceptibility to TMP-SMZ (94%) in community-acquired MRSA (CA-MRSA) isolates (Chen et al., 2006). In another study, all 320 isolates of MRSA (97% of USA300 strain) from outpatients with SSTIs were susceptible to TMP/SMZ (Moran et al., 2006). But this drug has also been considered to favor the selection and spread of resistant microorganisms on a population level. One study found that the mechanism was the selection for a class 1 integron, resulting in MDR Enterobacteriaceae in the intestinal flora of children (Van der Veen et al., 2009). Knowing that integrons are transferable genetic elements capable of expressing multiple resistance genes, the use of TMP-SMZ should be cautiously monitored regarding this potential collateral damage.

### Tetracyclines

Chlortetracycline, from which tetracycline was derived in 1953, is produced by *Streptomyces aureofaciens*. In the late 1960s, the second-generation long-acting compounds doxycycline (in 1966) and minocycline (in 1967) were semi-synthetically derived and commercialized. Tetracyclines are a class of broad-spectrum bacteriostatic antibiotics active against Gram-positive and Gram-negative bacteria, as well as against intracellular organisms. These characteristics, together with the low cost and rarity of major side effects, have made tetracyclines a widely used class of antibiotics (Nelson and Levy, 2011). However, it should be noted that these drugs are contraindicated in pregnant woman, neonates, and young children because of their particular effects on skeletal growth and dentition (Demers et al., 1968).

Tetracyclines have been evaluated as an effective oral treatment option for patients with SSTIs caused by MRSA (Ruhe and Menon, 2007). Minocycline, associated with surgical drainage, resulted in rapid resolution of MRSA-associated cutaneous abscesses (Cunha, 2013). Doxycycline has intrinsic activity against enterococci, including VRE, and has been stated as an option for oral treatment of VRE cystitis (Heintz et al., 2010). Bacteremia caused by VRE has been successfully treated with doxycycline (Moreno et al., 1994).

Recently, a MDR strain of CA-MRSA genotype USA300 has emerged, with plasmids that confer resistance to tetracycline, macrolides, and clindamycin (Diep et al., 2008). Staphylococcal resistance to tetracyclines is mainly conferred by active reflux, which is mediated by plasmid-located *tetK* and *tetL* genes, and ribosomal protection, which is mediated by chromosomal or transposonal *tetM* or *tetO* genes (Trzcinski et al., 2000). Studies relating the MICs of tetracycline, doxycycline, and minocycline to the presence of these genes suggest a crossresistance between doxycycline and tetracycline in all strains of *S. aureus* whereas minocycline could be in few cases spared. Caution is therefore recommended in the use of tetracyclines, given its ability to induce resistance in areas in which strains carry the inducible resistance genes (Schwartz et al., 2009). Besides, long-term use of tetracyclines in veterinary medicine has been associated with resistance emergence in commensal *Escherichia coli* in veal calves (Bosman et al., 2014).

### Chloramphenicol

Chloramphenicol, the active compound of which is produced by *Streptomyces venezuelae*, inhibits protein synthesis by binding reversibly to the 50S subunit of the bacterial ribosome. It is metabolized primarily in the liver. Dose adjustment is thus required in patients with hepatic insufficiency. Furthermore, chloramphenicol has good oral bioavailability and excellent tissue penetration. Its spectrum of activity is broad, including Gram-positive and Gram-negative bacteria, anaerobes, spirochetes, rickettsiae, chlamydiae, and mycoplasma. But, soon after chloramphenicol was released in the United States in 1949, reports linked this drug to rare but potentially lethal hematological side effects that restricted its use as last-resort therapy. However, as it is readily available and inexpensive, it is still used in many resource-limited settings (Falagas and Kopterides, 2007). Further studies are needed to update its toxicity risk with more accurate dosing and monitoring (Lautenbach et al., 1998).

Chloramphenicol was found to be effective against vancomycin-resistant *Enterococcus faecium* (VRE*f*), with bacteriostatic activity (Norris et al., 1995). A retrospective analysis of the outcomes of six patients with bacteremia caused by VRE*f* also showed that this agent was effective (Ricaurte et al., 2001). Several anecdotic reports of effective treatment with chloramphenicol in MDR bacterial severe infections been reported (Table 2).

Of 697 VRE samples from 28 US medical centers, only 2.4% were resistant to chloramphenicol (Zhanel et al., 2003). A significant decrease in rates of resistance to chloramphenicol among isolates of enterococci was reported from 2006 to 2009 in a Brazilian monocentric survey (Conceição et al., 2011). According to the SENTRY Antimicrobial Surveillance Program, the rate of chloramphenicol resistance among *E. faecalis* is greater in North America than in Europe (28.6 vs. 7.1%, respectively), but the reverse is true of *E. faecium* (0.5 and 15%, the latter due to clonal occurrences) (Deshpande et al., 2007). Chloramphenicol use for the treatment of VRE infections should take into account the potential for the development of resistance (Lautenbach et al., 2004). Indeed, high levels of resistance likely due to the widespread and unregulated use in resource-limited settings have been reported, particularly for the treatment of severe disease caused by MDR *Salmonella* sp. or VRE*f*. Most resistance mechanisms are efflux pumps such as *floR* and *cmlA*, as well as inactivating enzymes such as chloramphenicol acetyl-transferase *cat1* (Frye and Jackson, 2013). Furthermore, the association between fluoroquinolone use and chloramphenicol-resistant VRE emergence is of concern, since fluoroquinolone use has dramatically increased in recent years (Gould et al., 2004).

### Clindamycin

Clindamycin is produced by chemical modification of lincomycin, which was isolated in 1962 from *Streptomyces lincolnensis* (McGehee et al., 1968). This drug works by binding to the 50S ribosomal subunit of rRNA and inhibiting the initiation of peptide-chain synthesis. Although concern about *Clostridium difficile* colitis has limited the use of clindamycin, it remains an important antibiotic in the treatment of severe anaerobic infections.

The main advantage of clindamycin is its potential anti-exotoxin activity in necrotizing Panton-Valentin leucocidin (PVL)-positive CA-MRSA-complicated pneumonia or SSTIs, and it is usually used with another anti-MRSA antibiotic (Hidron et al., 2009). Clindamycin is also recommended by clinical practice guidelines as monotherapy for CA-MRSA SSTIs (Stevens et al., 2014).

Among 320 isolates of MRSA (97% of USA300 strain) from outpatients with SSTIs, 95% were susceptible to clindamycin (Moran et al., 2006). In another study, 98% of CA-MRSA isolates from patients with SSTI, were susceptible to clindamycin (Ruhe et al., 2007). Conversely, in Taiwan, a recent study found that nearly 96% of MRSA isolates from patients with necrotizing fasciitis were resistant to clindamycin. In a recent microbiological survey of CA-MRSA, 72 isolates (85% of European ST80 clone) from children with severe infections in Greece, clindamycin resistance increased significantly from 0% (2003) to 31.2% (2009) among the CA-MRSA isolates (Katopodis et al., 2010). This increase suggests clonal diversity in clindamycin susceptibility among MRSA strains that calls for direct surveillance and reports (Changchien et al., 2011). Moreover, inducible clindamycin resistance should be ruled out by a D-test because a substantial proportion of erythromycin-resistant isolates have shown clindamycin resistance despite appearing susceptible on initial testing (Siberry et al., 2003). Indeed, resistance genes confer resistance to macrolides, lincosamides, streptogramins (MLS). They cause resistance mainly by (i) target site modification, (ii) efflux pumps, and (iii) enzymatic inactivation. But new conjugative transposons (mobile genetic elements) carrying MLS genes along with other antibiotic resistance genes have recently been identified favoring the horizontal spread of resistance (Roberts, 2008). Thus, clindamycin should be used in combination with another anti-staphylococcal antibiotic as empiric therapy in patients with suspected severe staphylococcal infections.

### Pristinamycin

Pristinamycin, derived from *Streptomyces pristinaespiralis*, is an oral streptogramin antibiotic made up of two synergistic but structurally unrelated components, pristinamycin IA and pristinamycin IIA. It was discovered over 50 years ago (Cooper et al., 2014).

Although the data are limited, pristinamycin is a well-tolerated and effective alternative for the treatment of BJI due to Gram-positive bacteria including MRSA and VRE (Dancer et al., 2003; Ng and Gosbell, 2005; Ruparelia et al., 2008; Reid et al., 2010). The extended use of pristinamycin for BJI and other infections requires further evaluation.

Many enterococcal species, including those that are vancomycin-resistant though not including *Enterococcus faecalis*, are also susceptible to pristinamycin (Collins et al., 1993). In France, resistance to pristinamycin has remained low over the last 40 years, and typical susceptibility rates amongst staphylococci are 98% in the community (Quentin et al., 2001) and 93% in hospitals (Leclercq et al., 2003). But, also concerned by MLS resistance genes, the use of pristinamycin could favor the horizontal spread of resistance.

### Rifampicin

Rifampicin is a semi synthetic compound derived from *Streptomyces mediterranei*. It was introduced in 1967 as major part of the anti-tuberculous treatment. Rifampicin has an excellent tissue penetration and a unique activity on bacteria in biofilms growing on the surface of prosthetic devices. Despite the excellent bactericidal activity and oral bioavailability, the rapid emergence of resistance in bacteria constitutes a major limitation and therefore rifampicin should be used in combination with other antimicrobial agents. The cause of rifampicin-resistant mutations within bacteria are due to alteration in the *rpoB* gene (Forrest and Tamura, 2010).

Despite the lack of a control group and the limited number of patients, colistin and rifampicin appeared to be an effective and safe combination therapy for severe infections caused by MDR *Acinetobacter baumanii* (Motaouakkil et al., 2006; Bassetti et al., 2008) or MDR *Pseudomonas aeruginosa* (Tascini et al., 2006). Rifampicin with fusidic acid or rifampicin with fluoroquinolones treatment has been shown to be effective, in combination with surgical debridement, on early prosthesis joint infections (PJI) caused by MRSA (Aboltins et al., 2007). But combination therapy with rifampicin for the treatment of severe MRSA infections needs further studies (Forrest and Tamura, 2010).

### Fusidic acid (FA)

Fusidic acid, which is derived from the fungus *Fusidium coccineum*, was introduced into clinical practice in 1962 (Godtfredsen et al., 1962). It inhibits polypeptide-chain elongation by binding to the ribosome elongation factor G (EF-G)–GDP complex. FA has excellent oral bioavailability and is metabolized and excreted by the liver. The action of FA is mainly bacteriostatic against Gram-positive bacteria, but this drug has bactericidal activity at higher concentrations. Notably, FA has only limited activity against streptococci and enterococci. It is highly protein-bound and has been shown to have good concentrations in soft tissue, bone and synovial fluid (Turnidge, 1999).

Although there have been no randomized controlled trials of FA as a treatment for BJIs due to MRSA, several case series have reported its effectiveness, mostly in combination with another oral antibiotic (Drancourt et al., 1997; Aboltins et al., 2007; Ferry et al., 2010).

Rates of resistance to FA are higher among coagulase-negative staphylococci (CoNS) than among *S. aureus* isolates (Pfaller et al., 2010). Although the rate of *in vitro* resistance to FA among *S. aureus* is less than 10% in most countries (Wang et al., 2012), there is also a significant trend toward increased FA resistance associated with its increased use (Castanheira et al., 2008). For instance, in Kuwait, the rate of FA resistance increased dramatically from 22% in 1994 to 92% in 2004 (Udo et al., 2006). High usage of topical fusidic acid has been associated with clonal spread of fusidic acid-resistant *Staphylococcus aureus* (Williamson et al., 2014). Key resistance determinants include mutations in the *fusA* gene, which encodes EF-G, and plasmid-mediated resistance (i.e., acquisition of resistance gene *fusB*) (Howden and Grayson, 2006). Therefore, this agent must be used in combination with rifampin or other agents to prevent the emergence of resistance.

## Forgotten antibiotics for multidrug-resistant *M. tuberculosis*

Tuberculosis strains classified as MDR are those resistant to at least the two most potent first-line antituberculosis drugs (i.e., isoniazid and rifampicin) (Bosman et al., 2006). Extensively drug-resistant (XDR) tuberculosis (TB) strains are resistant to isoniazid or rifampicin (like MDR-TB), any fluoroquinolone, and at least one of three second-line injectable antituberculosis drugs (i.e., capreomycin, kanamycin, and amikacin) (Caminero et al., 2010). MDR and XDR-TB are generally thought to have high mortality rates. Treatment is challenging because of the high toxicity of second-line drugs and the longer treatment duration that participates to reduce the choice of available therapy. In a recent series of patients with XDR-TB, 58% experienced drug-associated adverse events including 9% lethal; therapy was discontinued in 28% (Shean et al., 2013). Right combination and rational use of available and safe antituberculosis drugs is therefore challenging (Mitnick et al., 2008). However, standardized strategies including pretreatment counseling, and follow-up are essential prerequisites in view to optimize therapy.

Here, we evaluated the clinical efficacy of 2 drugs belonging to group five in the World Health Organization (WHO) guidelines on MDR-TB treatment (i.e., clofazimine and amoxicillinie with clavulanate) and 2 antibiotics that are not listed but that may be candidates for evaluation against *Mycobacterium tuberculosis* (i.e., TMP/SMX and minocycline).

### Clofazimine

Clofazimine, a lipophilic riminophenazine antibiotic, was first synthesized in 1954 by Barry et al. as an anti-tuberculosis drug (Barry et al., 1957) but has known extended clinical use in the early 1970s to fight the emergence of *Mycobacterium leprae* resistant to sulfones.

In a report from Bangladesh, regimens containing clofazimine showed high efficacy (87.9% cure rate) in the treatment of MDR-TB (Van Deun et al., 2010). In a Korean study, 32 patients with MDR-TB and additional resistance to ofloxacin (11 with XDR-TB) were treated with clofazimine-containing regimens, with a lower cure rate (48.4%) (Yoo et al., 2013). Another recent report showed a cure rate of 38.4% with combination regimens including clofazimine for the treatment of MDR-TB, with mild adverse events (Xu et al., 2012). Adequate dose management would help to control adverse events, especially photosensitivity and gastric intolerance. Clofazimine's low cost is an additional advantage, although the drug is not widely available (Caminero et al., 2010).

### Amoxicillin with clavulanate

Amoxicilline-clavulanate, combined with other second-line drugs, has been successfully used to treat patients with MDR-tuberculosis (Nadler et al., 1991; Mitnick et al., 2008). The good tolerability, low price, and low toxicity profile of this drug have made it the drug of choice within group five according to the WHO guidelines for MDR-TB treatment (Bosman et al., 2006).

### Trimethoprim-sulfamethoxazole (TMP/SMX)

Between the late 1930s and the early 1950s, sulfonamides were used, usually as a monotherapy, for the treatment of tuberculosis. Because of the toxicity of the early sulfonamides and the introduction of highly effective anti-tuberculosis drugs such as isoniazid and streptomycin, both groups of drugs were abandoned for the treatment of tuberculosis in the early 1950s, and their use was essentially forgotten (Forgacs et al., 2009).

Clinical experience with TMP/SMX is scarce, but there is promising evidence supporting the treatment of XDR-TB with combination regimens (Forgacs et al., 2009; Dubourg et al., 2013). In light of those results and the fact that MDR and XDR *M. tuberculosis* strains have been found to be susceptible *in vitro* to both sulfadiazine and TMP/SMX (Ameen and Drancourt, 2013), further clinical trials are needed to consider sulfamides as alternative anti-tuberculosis antibiotics in combination regimens.

The great susceptibility of *M. tuberculosis* to TMP/SMX has been recently confirmed and attributed to the sulfonamide compound alone (Huang et al., 2012). In this study, susceptibility to sulfamethoxazole remained constant over a 12-year period despite the expanded use of TMP/SMX in the HIV-positive population.

### Minocycline

Minocycline activity against *M. tuberculosis* was first reported in 1983 (Tsukamura et al., 1983). This molecule is effective against *M. leprae* and has been used in combination regimens for the treatment of leprosy; however, minocycline was successfully used in the rescue treatment of an XDR-TB patient in Japan (Kawada et al., 2008). More recently, Brouqui et al. reported the first effective treatment with a regimen including linezolid, minocycline, sulfadiazine, and clofazimine according to the susceptibility profile of a patient with XDR-TB (Brouqui et al., 2013).

## Conclusion

In an era of increasing emergence of drug resistance and a scarcity of new antibiotics, there is a growing need to optimize the use of old and new antibiotics to treat infections. The arsenal of available antimicrobial drugs may be regulated by principles other than the lack of profit for drugs in limited-market areas. Indeed, in the last 20 years, pharmaceutical companies have developed highly profitable drugs that treat chronic diseases (i.e., hypertension, diabetes, dyslipidemia, and psychiatric disorders) rather than new antibiotics (Spellberg et al., 2004). Most likely for the same reasons, small countries have undergone shortages and market withdrawals of older antibiotics (Pulcini et al., 2012). Despite the increasing evidence of their effectiveness and safety, the United States Food and Drug Administration (FDA) has still not approved the use of some old antibiotics (i.e., fusidic acid, intravenous fosfomycin, pivmecillinam, temocillin, pristinamycin) (Table 3) http://www.accessdata.fda.gov/scripts/cder/drugsatfda/. As public health concern, cost effectiveness might be integrated in further comparisons between old and currently used antibiotics.

**Table 3:** **Availability from usual marketing system of the selected antibiotics in Europe, the USA, Canada and Australia (adapted from Pulcini et al., 2012)**.

**Molecules**	**Countries**
Colistin	AT, AU, BG, CA, CH, CZ, DK, EL, ES, FI, FR, FY, HU, IE, IT, LU, LV, MT, PL, PT, RO, SK, TR, UK, US
Fosfomycin IV	AT, DE, EL, ES, FR, IT
Fosfomycin PO	AT, BE, BG, CA, CH, DE, ES, FR, IE, IT, LU, NL, PL, PT, RO, SK, TR, US
Mecillinam	DK, EL
Pivmecillinam	AT, CA, DK, FI, FR, IS, NO, PT, SE, UK, ES, LU
Temocillin	BE, UK
Nitrofurantoin	AL, AT, AU, BE, BG, CA, CH, DE, DK, EE, EL, ES, FI, FR, HR, HU, IE, IS, IT, LT, LU, LV, MT, NL, NO, PL, PT, RO, TR, UK, US, XZ
Trimethoprim-sulfamethoxazole	AT, AU, BE, CH, CZ, DE, DK, EL, ES, FI, FR, HU, IE, IT, LU, NL, NO, PL, PT, RO, SE, SK, UK, US
Doxycycline	AT, AU, BE, CH, CZ, DE, DK, EL, ES, FI, FR, HU, IE, IT, LU, NL, NO, PL, PT, RO, SE, SK, UK, US
Minocycline	AT, AU, BE, CH, CZ, DE, EL, ES, FR, IE, IT, LU, NL, PT, RO, SE, UK, US
Chloramphenicol	AL, BG, CA, CZ, EL, ES, FI, IS, IT, MT, NO, PT, RS, SE, SK, TR, UK, US, XZ
Clindamycin	AT, AU, CH, DE, DK, EL, ES, FI, FR, HU, IE, IT, LU, NL, NO, PL, PT, RO, SE, SK, UK, US
Pristinamycin	FR, LU
Clofazimine	AU, CH, CZ, EL, ES, IE, FR, NL, US, UK

*AL, Albania; AT, Austria; AU, Australia; BE, Belgium; BG, Bulgaria; CA, Canada; CH, Switzerland; CZ, Czech Republic; DE, Germany; DK, Denmark; EE, Estonia; EL, Greece; ES, Spain; FI, Finland; FR, France; FY, Republic of Macedonia; HR, Croatia; HU, Hungary; IE, Ireland; IS, Iceland; IT, Italy; LT, Lithuania; LU, Luxembourg; LV, Latvia; MT, Malta; NL, Netherlands; NO, Norway; PL, Poland; PT, Portugal; RO, Romania; RS, Serbia; SE, Sweden; SI, Slovenia; SK, Slovakia; TR, Turkey; UK, United Kingdom; US, United States; XZ, Kosovo; IV, Intra-venous; PO, Per os*.

Optimization of antibiotic therapy in terms of pharmacokinetics and pharmacodynamics is needed to improve therapeutic outcomes but minimize the toxicity and the risk of resistance emerging during treatment (Mouton et al., 2011). Indeed, sub-therapeutic concentrations may favor the development of resistance in both the infecting pathogen and the commensal flora (Mohamed et al., 2012). Moreover, dosage adjustments, avoidance of co-administration of other toxic agents, and prompt discontinuation after early signs of toxicity are critical for the clinical revaluation of old antibiotics. For instance, recent studies revealed that adverse events with colistin were not as frequent as previously reported, in part due to more accurate prescription (Falagas and Kasiakou, 2006). Updates of old antibiotics toxicity assessment with optimized dosing and monitoring and adapted pharmacological studies are urgently warranted.

Finally, as old antibiotics are rarely included in surveillance programs, data regarding resistance rates, minimal inhibitory concentration (MIC), and harmonization are lacking (Mouton et al., 2011). Moreover, geographic variations make the extrapolation of local results inaccurate (Deshpande et al., 2007). Thus, susceptibility testing for old but effective and safe antibiotics may be integrated into routine practice in the way to ensure antimicrobial resistance local surveillance. As some of these old antibiotics (i.e., fosfomycin, TMP/SMX, clindamycin, pristinamycin, and fusidic acid) could also favor horizontal spread of resistance, their reuse should be associated with a global ecology survey. In this case, combination therapy remains an alternative for their reuse.

In summary, all old antibiotics included in this review have been associated with successful treatment of MDR bacterial infections. Their reuse represents a promising strategy to fight antimicrobial resistance. But some points remain critical for their revival in a sustainable manner and need further evaluations. First, clinical trials comparing the clinical, safety, and cost effectiveness with current antibiotics are lacking. Second, prescription of old antibiotics needs also to be regulated by antibiotic stewardships and guided by resistance rates monitoring. Third, optimization of the usage of old antibiotics remains a priority that may be considered of similar importance to that of the assessment of new drugs.

## Author contributions

Nadim Cassir wrote the paper. Jean-Marc Rolain and Philippe Brouqui reviewed and edited the paper for content and accuracy.

### Conflict of interest statement

The authors declare that the research was conducted in the absence of any commercial or financial relationships that could be construed as a potential conflict of interest.
